# Mayer-Rokitansky-Küster-Hauser Syndrome Associated With Diabetes Mellitus and Renal Anomalies in an Adolescent Girl: A Rare Case Report

**DOI:** 10.1016/j.aed.2025.10.009

**Published:** 2025-10-15

**Authors:** Manar F. AlShammari, Saad M. AlShammari

**Affiliations:** 1 Consultant Family Medicine and Diabetologist, Program Director of the Family Medicine Residency Program, Royal Commission Hospital, Jubail, Saudi Arabia; 2 Consultant Orthopedic Surgeon, Program Director of the Orthopedic Residency Program, Armed Forces Hospital Dhahran, Ministry of Defense, Saudi Arabia

**Keywords:** MRKH syndrome, primary amenorrhea, diabetes mellitus, MURCS association, congenital anomalies, monogenic diabetes, Renal Anomalies, MODY5

## Abstract

**Background/Objective:**

Mayer-Rokitansky-Küster-Hauser (MRKH) syndrome is a rare congenital anomaly characterized by agenesis or hypoplasia of the uterus and upper vagina in phenotypically normal females. Although patients with MRKH syndrome typically exhibit normal secondary sexual development, associations with extragenital anomalies and metabolic conditions such as diabetes mellitus have been increasingly reported.

**Case Description:**

A female patient initially presented at the age of 15 (currently 19 year old) with primary amenorrhea and absence of secondary sexual characteristics. Initial laboratory evaluation showed elevated follicle-stimulating hormone (FSH) and thyroid-stimulating hormone (TSH) levels, while thyroid antibodies and prolactin were within normal limits. Pelvic magnetic resonance imaging revealed poorly visualized ovaries, a hypoplastic or absent uterus and upper vagina, and urinary tract anomalies including a duplicated right ureter. One year later, she developed symptoms suggestive of diabetes mellitus and was found to have elevated random and fasting blood glucose levels. Her mother had been diagnosed with type 2 diabetes mellitus prior to conception. The patient was referred to endocrinology to evaluate the possibility of monogenic diabetes. The constellation of uterovaginal aplasia, renal anomalies, and endocrine dysfunction raises clinical suspicion of an expanded phenotypic spectrum or a potential overlap syndrome, such as Müllerian duct aplasia, renal dysplasia, and cervicothoracic somite anomalies (Reference ranges: FSH: 3-10 mIU/mL in follicular phase; TSH: 0.4-4.0 μIU/mL).

**Conclusion:**

This case highlights the importance of comprehensive systemic evaluation in patients with MRKH syndrome, especially when extragenital anomalies or metabolic abnormalities such as diabetes mellitus coexist. Early recognition and multidisciplinary management are essential for optimal care and may provide valuable insights into potentially shared developmental and genetic pathways.

**Highlights**

• Mayer-Rokitansky-Küster-Hauser (MRKH) syndrome can rarely coexist with renal anomalies and metabolic disorders such as diabetes mellitus, broadening its phenotypic spectrum
• Comprehensive systemic evaluation is essential in patients with MRKH syndrome who present with extragenital anomalies or endocrine abnormalities
• Early referral for genetic testing, including evaluation for HNF1B mutations, should be considered when MRKH is associated with renal or metabolic manifestations
• Clinical management requires a multidisciplinary approach involving endocrinology, gynecology, urology, and genetics to optimize outcomes
• Recognition of atypical presentations, such as absent secondary sexual characteristics with early-onset diabetes, may provide new insights into possible genetic overlap syndromes

**Clinical Relevance**

This case demonstrates the unusual coexistence of Mayer-Rokitansky-Küster-Hauser syndrome, renal anomalies, and early-onset diabetes mellitus, emphasizing the importance of multidisciplinary evaluation and consideration of genetic testing in atypical clinical presentations.

## Introduction

Mayer-Rokitansky-Küster-Hauser (MRKH) syndrome is a rare congenital disorder of the female reproductive tract, characterized by agenesis or severe hypoplasia of the uterus and the upper two-thirds of the vagina in females with a normal 46,XX karyotype and normal ovarian function. Despite normal external genitalia and typical secondary sexual development, patients present with primary amenorrhea during adolescence. The estimated incidence of MRKH syndrome is approximately 1 in 4500 to 5000 female births.^1^

The syndrome exists in 2 major forms: Type I (isolated Müllerian agenesis) and Type II (associated with extragenital malformations such as renal, vertebral, or auditory anomalies). A more complex variant, Müllerian duct aplasia, renal dysplasia, and cervicothoracic somite anomalies association, has also been described.^2^

This case is particularly unusual due to the coexistence of MRKH syndrome with diabetes mellitus and urinary tract abnormalities, including ureteral duplication. The association between MRKH syndrome and metabolic disorders, such as diabetes, is rarely reported in the literature, and its pathophysiological basis remains unclear. Such cases raise the possibility of a broader syndromic or genetic spectrum underlying MRKH syndrome.^3^

To our knowledge, the simultaneous occurrence of MRKH, renal anomalies, and suspected MODY is extremely rare and underreported. This case aims to contribute to the limited literature exploring syndromic associations between MRKH and early-onset diabetes mellitus.

## Case Description

A 15-year-old female (currently aged 19) presented to the family medicine clinic with a chief complaint of primary amenorrhea and the absence of secondary sexual characteristics. Her menstrual history revealed only 1 episode of reddish vaginal spotting 2 years earlier, which lasted for a single day. She denied symptoms of hirsutism, galactorrhea, visual disturbances, anosmia, or cyclic pelvic pain. There was no history of excessive exercise, psychiatric illness, or use of hormonal medications.

The patient was born at term via normal vaginal delivery of consanguineous marriage, with a neonatal history of jaundice that required no intervention. Growth and developmental milestones were within normal limits, and she had no surgical history. Family history revealed that her older sister had also experienced amenorrhea, though no definitive diagnosis was established. Her mother had been diagnosed with type 2 diabetes mellitus 1 year before conceiving the patient, and both her maternal and paternal grandfathers were diabetic.

Anthropometric measurements, including BMI of 27.4 kg/m^2^, were within normal limits. There was no evidence of acanthosis nigricans or other dysmorphic features.

Laboratory investigations revealed markedly elevated FSH at 63.54 mIU/mL (reference: 3.5-12.5 mIU/mL in the follicular phase) and TSH at 8.14 μIU/mL, rising to 13.33 μIU/mL on repeat testing (reference: 0.48-4.17 μIU/mL). Prolactin was normal at 6.22 ng/mL (reference: 3-14.4 ng/mL). Free T4 was 1.09 to 1.26 ng/dL (reference: 0.83-1.43 ng/dL), and free T3 was 3.0 to 3.18 pg/mL (reference: 2.3-4.2 pg/mL), both within normal ranges. Thyroid peroxidase antibody was 11.63 IU/mL (reference: <26 IU/mL), and anti-thyroglobulin antibody was 15.69 IU/mL (reference: <64 IU/mL), also within normal limits. Based on these findings, she was diagnosed with subclinical hypothyroidism and started on levothyroxine therapy. Chromosomal analysis revealed a normal female karyotype (46,XX) without numerical or structural abnormalities (reference ranges: FSH: 3-10 mIU/mL in the follicular phase; TSH: 0.4-4.0 μIU/mL).

**Fig. 1:**
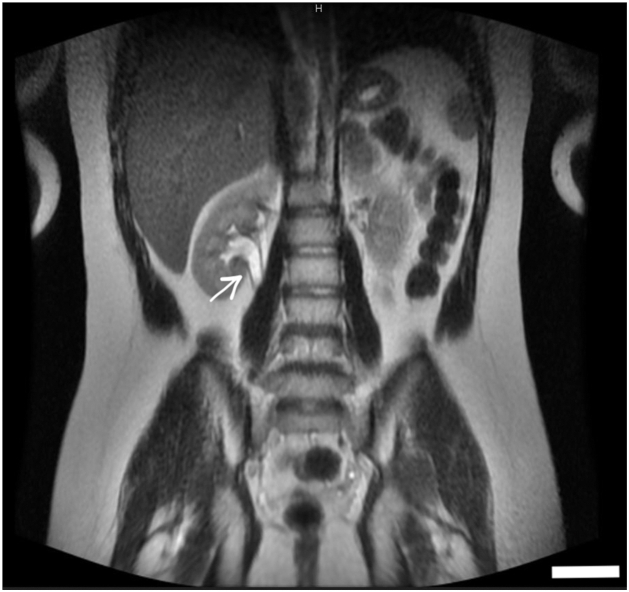
Coronal T2-weighted MRI demonstrating a duplicated *Right* ureter and double collecting system (arrow) with mild dilatation of the *Lower* moiety. The kidneys are in normal anatomical position and size with no evidence of mass or nephrolithiasis.

**Fig. 2:**
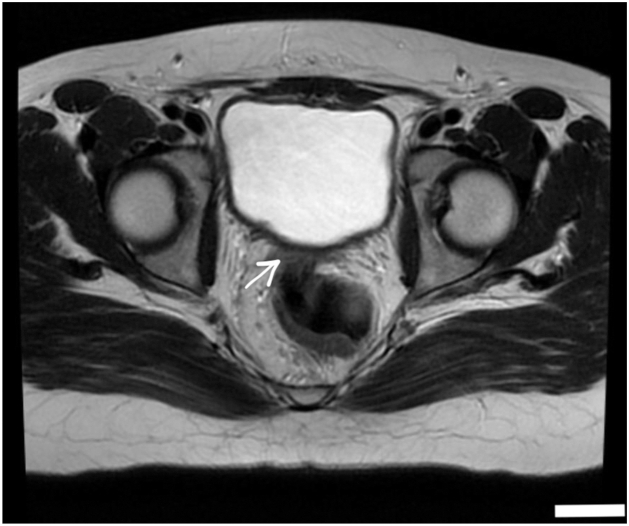
Axial pelvic MRI image showing an absent uterus (arrow) located posterior to the bladder. Ovaries are not visualized. The urinary bladder is distended with a normal wall and adjacent musculature. No pelvic mass was detected.

**Fig. 3:**
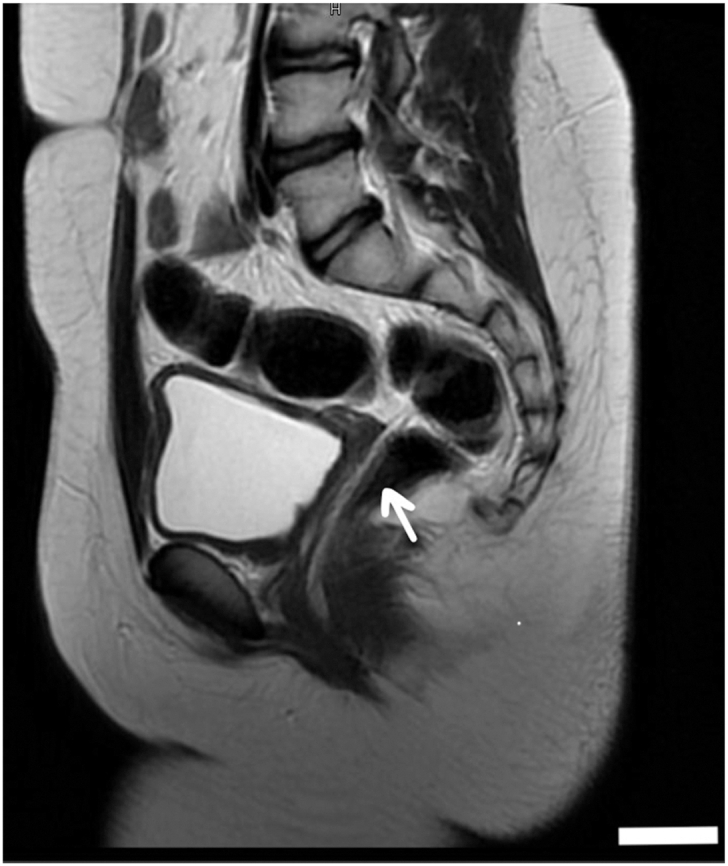
Sagittal T2-weighted MRI demonstrating an absent or hypoplastic uterus and *Upper* vagina (arrow), located posterior to the bladder.The bladder appears normal in size and shape. The bowel loops and spine alignment are unremarkable.

Pelvic magnetic resonance imaging (MRI) demonstrated a right-sided rudimentary uterine bud, poorly visualized bilateral ovaries that appeared atrophic, and a right renal double collecting system with duplicated ureters and mild dilatation of the lower moiety (Fig. 1, Fig. 2, Fig. 3). Based on imaging findings and endocrine profile, a diagnosis of MRKH syndrome type II with urogenital anomalies was established. Urology consultation was requested for renal evaluation.

She reported no urological symptoms. Renal ultrasound demonstrated a right renal double collecting system with double ureters and mild dilatation of the lower moiety. Otherwise, both kidneys are average sized with normal anatomical position. Renal sinus and cortical echogenicity were within normal limits bilaterally and there is no evidence of nephrolithiasis or renal mass bilaterally. The urinary bladder is distended with average wall thickness, no stones or masses. Also, unremarkable urinalysis and creatinine test.

One year later, the patient presented with a 2-week history of headache, fatigue, polyuria, and polydipsia. Home glucose monitoring showed random blood glucose level exceeding 300 mg/dL. She had no history of diabetic ketoacidosis or prior hypoglycemic/hyperglycemic emergencies. In-clinic fasting glucose was 126 mg/dL. Further testing included C-peptide and HbA1c levels to classify the type of diabetes, as summarized in Table 1. Her HbA1c was 9.76% with normal C-peptide (2.17 ng/mL) and insulin (13.73 μU/mL). Urinalysis revealed glycosuria with negative ketones. Insulin autoantibodies (IAA), glutamic acid decarboxylase antibodies (GAD65), and anti-Islet cell antibodies requested and were negative.

The patient and her guardian initially refused pharmacologic treatment but later consented to initiate metformin and sitagliptin. The overall clinical progression is outlined in Table 2. She was referred to endocrinology for genetic evaluation to rule out possible monogenic diabetes, such as MODY, given the early onset and family history of diabetes and congenital anomalies.

**Table 1:** Summary of Diabetes-Related Laboratory Results

Test	Result	Reference	Comment
Random glucose	>300 mg/dL (home); 8.72 mmol/L (∼157 mg/dL)	<11.1 mmol/L (<200 mg/dL)	Elevated
Fasting glucose	126 mg/dL (8.4 mmol/L)	70-99 mg/dL (4.1–5.9 mmol/L)	High
HbA1c	9.76%	4-6%	Very high, diagnostic
C-peptide	2.17 ng/mL	0.8-3.1 ng/mL	Within normal range
Insulin	13.73 μU/mL	2-25 μU/mL	Within normal range
GAD65, IAA, ICA	Negative	Negative	Absence reduces likelihood of type 1 DM but does not exclude it
Urinalysis	Glycosuria, ketone negative	—	Non-ketotic hyperglycemia

**Table 2:** Clinical Timeline

Clinical Event	Age/Date	Notes
Onset of primary amenorrhea	Age 15	No development of secondary sexual characteristics
Initial evaluation	Age 15	Elevated FSH and TSH, absent uterus and ovaries, duplicated right ureter
Subclinical hypothyroidism diagnosis	Age 15	Started Levothyroxine
Diagnosis of MRKH Type II	Age 15	Based on imaging and lab findings
Onset of diabetic symptoms	Age 16	Headache, thirst, polyuria, fatigue
Diabetes confirmation	Age 16	HbA1c 9.76%, normal C-peptide, negative autoantibodies
Referral for MODY genetic testing	Age 19	Pending/Completed/Not available

## Discussion

Mayer-Rokitansky-Küster-Hauser (MRKH) syndrome is a rare developmental anomaly of the Müllerian ducts, most commonly presenting as primary amenorrhea in adolescent females with normal secondary sexual characteristics and karyotype (46,XX).^1^ The presented case deviates from the typical clinical picture in 2 significant ways: the absence of secondary sexual characteristics and the coexistence of type 2 diabetes mellitus—features that are rarely reported together in the literature.^1^

Several studies have reported the association of MRKH syndrome with extragenital malformations, including renal and skeletal anomalies, especially in MRKH type II and Müllerian duct aplasia, renal dysplasia, and cervicothoracic somite anomalies association.^2^ In our case, the presence of a duplicated right ureter and renal collection confirms urogenital tract involvement, consistent with MRKH type II. However, the absence of identifiable ovaries on imaging, along with lack of secondary sexual development and elevated FSH, suggests a possible overlap with primary ovarian insufficiency or gonadal dysgenesis, making the diagnostic process more complex.^2^^,^^4^^,^^5^

No definitive conclusion can be drawn without genetic testing.

This case was diagnostically challenging due to the atypical endocrine profile, non-visualization of ovaries, and delayed metabolic presentation. A multidisciplinary approach involving endocrinology, gynecology, urology, and genetics was essential in reaching a comprehensive diagnosis and initiating appropriate management.

At the time of reporting, genetic testing results for monogenic diabetes (MODY) were still pending. However, despite multiple reminders to the patient and her mother, they did not attend the appointment for genetic sampling. Therefore, testing could not be completed. Nevertheless, the early-onset, absence of autoantibodies, and positive family history make MODY a strong consideration. If confirmed, it would further expand the clinical spectrum of associated anomalies in MRKH syndrome.^6^

Lessons learned from this case highlight the importance of systemic evaluation in patients diagnosed with MRKH, particularly when clinical features extend beyond the reproductive system. Clinicians should maintain a high index of suspicion for endocrine and renal anomalies, and consider early genetic consultation when the phenotype deviates from classical MRKH presentation.

Such cases underscore the need for expanding genotype-phenotype correlation studies in congenital and metabolic disorders.

### Learning Points

1. A multidisciplinary approach improves diagnostic accuracy and patient outcomes, especially in complex or atypical presentations of congenital syndromes.
2. Genetic testing is essential in atypical MRKH presentations to confirm diagnosis, guide management, and support family counseling.
3. Early recognition of renal and metabolic anomalies (such as duplicated ureters and early-onset diabetes) is critical for providing comprehensive care in MRKH patients.
4. Emerging evidence suggests that mutations in genes such as HNF1B may contribute to both MRKH syndrome and forms of monogenic diabetes [e.g., MODY5], but this association requires further confirmation and should not be assumed in individual cases.
5. MRKH syndrome can occasionally present with atypical features, such as absence of secondary sexual characteristics and endocrine abnormalities, suggesting a broader phenotypic or syndromic spectrum.

## Limitations

A notable limitation of this case report is the lack of confirmatory genetic testing results at the time of submission. Although the clinical presentation is highly suggestive of a monogenic form of diabetes, particularly MODY, the patient’s genetic sequencing—specifically for HNF1B and other related mutations—was requested but could not be completed. Despite multiple reminders, the patient and her mother did not attend the scheduled appointment for genetic sampling. However, this limitation does not significantly weaken the case report; rather, the availability of genetic confirmation would serve as an added strength, providing further molecular insight into the suspected syndromic overlap.

Another limitation is the inability to perform full gynecological examination due to patient refusal, which may have limited clinical confirmation of internal anatomy.

## Conclusion

This case illustrates the clinical variability of MRKH syndrome when associated with renal and metabolic anomalies. The coexistence of uterovaginal agenesis, renal duplication, and early-onset diabetes suggests a potential syndromic overlap, possibly linked to HNF1B mutations. Early genetic testing and multidisciplinary evaluation are essential to ensure accurate diagnosis, guide management, and support family counseling.

No definitive conclusion can be drawn without genetic testing.

## Conflicts of interest

None disclosed.
